# Hydroxyl Groups Induce Bioactivity in Silica/Chitosan Aerogels Designed for Bone Tissue Engineering. In Vitro Model for the Assessment of Osteoblasts Behavior

**DOI:** 10.3390/polym12122802

**Published:** 2020-11-26

**Authors:** Antonio Perez-Moreno, María de las Virtudes Reyes-Peces, Deseada María de los Santos, Gonzalo Pinaglia-Tobaruela, Emilio de la Orden, José Ignacio Vilches-Pérez, Mercedes Salido, Manuel Piñero, Nicolás de la Rosa-Fox

**Affiliations:** 1 Instituto de Investigación e Innovación Biomédica de Cádiz (INIBICA), 11009 Cádiz, Spain; maria.reyes@uca.es (M.d.l.V.R.-P.); emilioi.delaorden@uca.es (E.d.l.O.); nachojivp@yahoo.es (J.I.V.-P.); mercedes.salido@uca.es (M.S.); manolo.piniero@uca.es (M.P.); nicolas.rosafox@uca.es (N.d.l.R.-F.); 2 Instituto de Microscopía Electrónica y Materiales (IMEYMAT), University of Cadiz, 11510 Cádiz, Spain; 3 Department of Condensed Matter Physics, Faculty of Science, University of Cadiz, 11510 Cádiz, Spain; 4 Department of Physical Chemistry, Faculty of Science, University of Cadiz, 11510 Cádiz, Spain; desire.delossantos@uca.es; 5 Department of Histology, SCIBM, Faculty of Medicine, University of Cadiz, 11004 Cádiz, Spain; gonzalo.pinaglia@hotmail.com

**Keywords:** aerogels, biomaterials, fracture toughness, hydroxyapatite (HAp), bone tissue engineering, osteoinduction

## Abstract

Silica (SiO_2_)/chitosan (CS) composite aerogels are bioactive when they are submerged in simulated body fluid (SBF), causing the formation of bone-like hydroxyapatite (HAp) layer. Silica-based hybrid aerogels improve the elastic behavior, and the combined CS modifies the network entanglement as a crosslinking biopolymer. Tetraethoxysilane (TEOS)/CS is used as network precursors by employing a sol-gel method assisted with high power ultrasound (600 W). Upon gelation and aging, gels are dried in supercritical CO_2_ to obtain monoliths. Thermograms provide information about the condensation of the remaining hydroxyl groups (400–700 °C). This step permits the evaluation of the hydroxyl group’s content of 2 to 5 OH nm^−2^. The formed Si-OH groups act as the inductor of apatite crystal nucleation in SBF. The N_2_ physisorption isotherms show a hysteresis loop of type H3, characteristic to good interconnected porosity, which facilitates both the bioactivity and the adhesion of osteoblasts cells. After two weeks of immersion in SBF, a layer of HAp microcrystals develops on the surface with a stoichiometric Ca/P molar ratio of 1.67 with spherulite morphology and uniform sizes of 6 μm. This fact asserts the bioactive behavior of these hybrid aerogels. Osteoblasts are cultured on the selected samples and immunolabeled for cytoskeletal and focal adhesion expression related to scaffold nanostructure and composition. The initial osteoconductive response observes points to a great potential of tissue engineering for the designed composite aerogels.

## 1. Introduction

Bone is the structural component of vertebrates. It is a hybrid material composed of a 45% inorganic network formed by nanocrystals of hydroxyapatite (HAp) and calcium carbonate (CaCO_3_) grown in 30% of collagen fibers as organic phase and 25% remaining as water [1]. These contents vary in the different bones (cortical and trabecular) and animals. Mimetization has always been an effective method to solve the difficulties of nature. These SiO_2_/CS aerogels have never been used in bone reparation. This study shows the potential of these aerogels to serve as a platform for the regeneration and repair of human tissues. The mechanical response of bone is also quite strain-rate sensitive. When the velocity of loading increases, both the elastic modulus and the fracture stress increase. Hence, the stiffness increases with the strain rate and, therefore, the hardness [2].

Regarding materials for bone regeneration, the first thing to obtain is a scaffold that is as similar as possible to the inorganic part of the bone. It must have the following properties [3]:

- Biocompatibility and bioactivity, understood as the capacity to support normal cellular activities, without the appearance of toxic effects (osteoconductive and osteoinductive).
- Mechanical properties compatible with the elastic behavior of the bone, with the capacity to withstand the various kinds of stresses to which the bone is subjected.
- A distribution of interconnected pores from mesopores to gigapores (2 nm–300 μm).
- Bioreabsorbability and/or biodegradability.

A wide range of materials is currently used for that purpose [4,5,6,7,8,9]. The present study is focused on the synthesis of a material composed of silica-chitosan-calcium carbonate, and the synthesis route will be the sol-gel method [10]. The use of solvent can be avoided if we subject the mixture to the action of high-power ultrasound [11,12]. A homogeneous and transparent solution that will gel in a short time is formed, resulting in a sonogel [13]. There are important differences between classic gels and sonogels; the latter are more dense, homogeneous, and have finer structures. The rupture of the gel during drying due to the capillary forces associated with the gas–liquid interface can be avoided by treating the gel in an autoclave under supercritical conditions of pressure and temperature for the solvent (CO_2_ in our case), taking care that during the treatment, no liquid-vapor equilibrium curve is crossed [14]. An aerogel is obtained, and a sonoaerogel is prepared with ultrasound.

The mesoporous silica-based gels are ideal for bone tissue regeneration due to their properties, such as [5,6,7,8] high specific surface area, controllable pore size (mesopores from 2 to 50 nm), and well-ordered structure (mesoporous channels). They are also ideal as starting materials for the manufacture of 3D scaffolds for bone regeneration [15,16,17,18,19,20,21,22]. These materials have also been shown to have zero or very low cytotoxicity in vitro and very good biodegradability in simulated biological serum [19]. Further, in mouse experiments, it has been shown that mesoporous silica is biocompatible, biodegradable, and bioexcretable [23,24]. Chitosan is a good biopolymer for the manufacture of 3D scaffolds for bone regeneration. It is biodegradable, biocompatible, antibacterial, acts as a wound healer, bioadhesive, etc. The main disadvantage is its mechanical properties: it does not support the load that it needs to support in order to replace the bone [17,23]. Finally, calcium carbonate is the source of calcium, which is necessary for a material to be bioactive [3].

Two objectives are achieved as a novelty in this study of SiO_2_/CS hybrids aerogels: first, control of the surface -OH groups that act as nucleation sites for the apatite crystal growth, improving the bioactivity, and second, an osteoconductive response that points to a great potential for tissue engineering of the human bone and connective soft tissues.

## 2. Materials and Methods

### 2.1. Materials

Chitosan (CS) (low molecular weight 50,000–190,000 Da, 75–85% deacetylated, CAS 9012-76-4) was purchased from Sigma-Aldrich (San Louis, MO, USA). Tetraethylortosilicate (TEOS, 99%, CAS 78-10-4,) was supplied by Alfa Aesar (Haverhill, MA, USA). Hydrochloric acid (37%, Pharma grade, CAS 7647-01-0) was obtained from Alfa Aesar. Absolute ethanol (99.5%, CAS 64-17-5) was purchased from Panreac (Barcelona, Spain). HOB^®^ human osteoblasts, fetal calf serum, and osteoblast growing medium (Promocell, Heidelberg, Germany). Paraformaldehyde, PBS, Triton x-100, bovine serum albumin, methanol rhodamine-phalloidin, and monoclonal anti-vinculin FITC conjugate were all purchased from Sigma-Aldrich. “Hard Set Vectashield with DAPI^®^ (Vector, Burlingame, CA, USA)”.

### 2.2. Aerogel Synthesis

**Figure 1:**
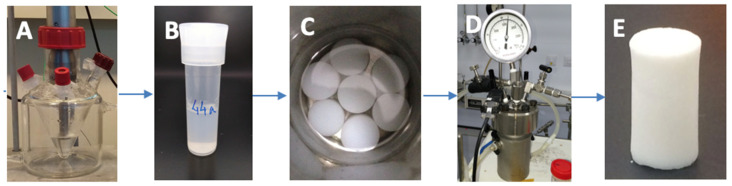
Sol-gel process assisted with high-power ultrasounds (**A**). Aging and solvent exchange in ethanol (**B**,**C**), Dried in an autoclave under supercritical conditions of CO_2_ (**D**). Monolithic aerogels were obtained (**E**).

The preparation procedure is given schematically in Figure 1. In a typical experiment, silica sol was prepared by mixing TEOS/water/HCl in a molar ratio of 1:4:0.05, under the action of ultrasound, by exposure to 4.5 kJ/cm^3^ of energy in a glass reactor—sol A. Separately, a CS solution was prepared by adding 2.5 g of low molecular weight CS (50,000–190,000 Da) and adding 0.6 M HCl to complete 250 mL of solution. A homogeneous mixture was obtained under the action of ultrasound by exposure to 10 kJ/cm^3^ of energy—sol B. Finally, the final solution was prepared by mixing sol A and sol B; all the reactants were subjected to a new dose of ultrasound (0.5 kJ/cm^3^) to get perfectly transparent solutions. The molar ratio of water/TEOS was kept constant at 30:1 in all samples. Sols were kept at 50 °C on a stove to gel for 24–48 h. SiO_2_/CS composites were synthesized and were nominated SCSX (Silica ChitoSan), X being the weight percentage of CS with respect to SiO_2_ (4, 8, 16, and 20 wt%, the real content is indicated in Table 1). Hybrid samples were then aged in an ethanol bath for 28 days in order to strengthen them and to obtain the maximum shrinkage before drying. Finally, the remaining water in the hybrid hydrogels, which would cause problems at the drying step, was removed by soaking in ethanol for another week with the daily exchange.

Conversion of hybrids to their respective aerogels was accomplished by supercritical (SC) CO_2_ drying as follows: cylindrical alcogels (10 mm diameter, 20 mm height) were introduced into a 100 mL autoclave and were covered with excess ethanol. Then, the vessel was cooled down to 4–5 °C and pressurized with liquid CO_2_ (6.5 MPa). When the interstitial solvent was considered to be totally extracted, the vessel was heated up to 40 °C at 1 °C/min, while the pressure rose to 10 MPa, in order to exceed the critical point of CO_2_ (31.06 °C and 7.4 MPa). Finally, the CO_2_ was isothermally vented out using a depressurization rate of 3 MPa/h.

### 2.3. Physicochemical Characterization

#### 2.3.1. Textural Characterization

The apparent density was obtained by measuring the mass with a microbalance (precision ± 0.1 mg) and the volume of the cylindrical samples with a slide caliper (accuracy 1/20 mm).

The textural characteristics of the hybrid aerogels were investigated using nitrogen physisorption experiments (Micromeritics ASAP2020, working at 77 K, and equipped with pressure transducer resolution of 10^−4^ mm Hg). Specific surface area, specific pore volume, and mean pore diameter were determined, considering standard models for the analysis (BET and BJH, respectively) [10]. Prior to these experiments, samples were milled in an agate mortar and degassed at 120 °C for 6 h.

Thermogravimetric analysis (TGA) was performed on a Perkin-Elmer TGA7 thermogravimetric analyzer (Madrid, Spain) at 10 °C min^−1^ and ramped from 50 to 900 °C under air atmosphere. The different weight loss steps were determined in order to evaluate the hydroxyl contents (–OH groups) for dehydration up to 200 °C and dehydroxylation from 400 to 700 °C.

#### 2.3.2. Structure Characterization

The study of the microstructural surface morphology was performed by scanning electron microscopy (SEM) in a NovaNanoSEM 450 model. The study of the microstructural surface morphology was performed by SEM with a thermionic filament emission (FEI Quanta; resolution: 3.5 nm) using the low-vacuum capability in the pressure range 10–130 Pa, which enables analysis of wet specimens without previous preparation. Energy-dispersive X-ray (EDX) spectroscopy was performed to evaluate the Ca/P molar ratio of the crystals.

The surface chemical structure was studied by Fourier transform infrared spectroscopy (FTIR). All the infrared spectra were collected at room temperature on a Fourier transform spectrometer Bruker Tensor 37, (Madrid, Spain) with a resolution of 4 cm^−1^ and 100 scans in the region 4000–400 cm^−1^. The samples were stored overnight in a stove at 60 °C, then ground and mixed with KBr and pressed into a self-supporting wafer.

### 2.4. Mechanical Characterization

Stress/strain curves by uniaxial compression were performed in a Shimadzu AG-I Autograph model equipped with load cells of 5 kN and 500 N. A crosshead speed of 0.1 mm min^−1^ was applied, which corresponded to a 2 × 10^−4^ s^−1^ strain rate; all tests were carried out until fracture. The mechanical parameters were obtained by unconfined compression of cylindrical specimens of 15–20 mm in height and 8–10 mm in diameter, fulfilling the ASTM D7012 standard (h = 2D). The compressive strength and deformation at fracture were obtained from the maximum stress and maximum deformation before sample failure. The elastic modulus was estimated from the slope of the stress–strain curve. All the experiments were performed in triplicate to get statistical significance.

### 2.5. Water Uptake

Water uptake ability M(t) was assessed by gravimetry according to Equation (1) by immersing samples in deionized water and weighing them before and after different immersion times (from t = 0 up to several days)

(1) $$M\left(t\right)=\frac{m\left(t\right)-m\left(0\right)}{m\left(0\right)}$$

where m(t) and m(0) represent the weight of wet and dry samples, respectively [15]. Before each measurement was made, the excess surface liquid was wiped out from the specimen, and the weight was recorded; the sample was then immediately returned to the bath. This process was repeated until no weight change was observed for several hours, at the end of which, the equilibrium of absorption was attained. The process was repeated with three different specimens of the same scaffold, and data were reported as mean and standard deviation.

### 2.6. Bioactivity Evaluation

In vitro bioactivity tests were carried out by soaking aerogel samples in simulated body fluid (SBF) at 37 °C for 7, 14, and 28 days. The SBF was prepared following the protocol described by Kokubo [23], and the volume of SBF needed for the tests was calculated from Equation (2):

(2) Vs= Sa10

where Vs is the volume of SBF (mL), and Sa is the apparent surface area of the specimen (mm^2^). As our material was porous, we must put more SBF until the sample was completely submerged.

All chemicals (KCl, NaCl, NaHCO_3_, CaCl_2_, K_2_HPO_4_·3H_2_O, MgCl_2_·6H_2_O, Na_2_SO_4_, NH_2_C(CH_2_OH)_3_ (2-Amino-2-hidroximetil-propano-1,3-diol), and HCl) were purchased from Sigma-Aldrich. After 0, 7, 14, and 28 days, the SBF was removed using filter paper. The samples were rinsed with distilled water, dehydrated in ethanol, and dried on a stove. The dried samples were analyzed by SEM and energy-dispersive spectroscopy (EDS).

### 2.7. Biocompatibility Tests In Vitro

HOB^®^ cells were seeded on the preselected scaffolds under sterile conditions. Cells were grown to confluence and then detached, counted to optimal cell density, and analyzed for cell viability under an automated Luna^®^ cell counter (Invitrogen, Carlsbad, CA, USA). Cells did not exceed ten population doublings. In order to achieve optimal sterilization, aerogels were sterilized in a clinically standardized autoclave prior to cell seeding. Once sterilized, the samples were placed under sterile conditions in a laminar flow chamber on Mattek^®^ glass bottom wells. A drop of 50 μL of cell suspension at a density of 15,000 HOB^®^ cells/cm^2^ was added to each sample and kept for 30 min under incubation in humid conditions at 37 °C and 5% CO_2_ to ensure optimal cell attachment and avoid dispersion. Wells were then filled with OGM^®^ (Oteoblast Gowth Medium) supplemented to a final concentration of 0.1 mL/mL of fetal calf serum at 37 °C and 5% CO_2_ and incubated during experimental times. The growth medium was changed every two days and collected for the determination of degradation products.

Live/dead cell assay was performed to evaluate the viability/cytotoxicity of HOB cells grown on the silica/chitosan aerogel. After being incubated for 24 h, 48 h, and 7 days, the cell/scaffold constructs were rinsed with PBS twice and then stained with calcein-AM (0.5 μL/mL) in PBS and ethidium homodimer-1 (EthD-1) (2 μL/mL) in PBS to display the live and dead cells, respectively. The cell/scaffold constructs were observed on the confocal laser scanning microscope. 

Scaffolds with on growing cells were immunolabeled after 24 h, 48 h, and 1 week with rhodamine-phalloidine and vinculin in order to assess cytoskeletal changes and focal adhesion development. At least five samples of each type were seeded and analyzed per experiment. The test groups were as follows: SCS8 and control. HOB^®^ cells grown on glass under conditions described above were used as control.

#### 2.7.1. Cell Morphology and Spreading

Cells were daily examined with the phase-contrast microscope in order to evaluate cell morphology, alignment distribution, and spreading. The initial adhesion phase to surfaces was assessed prior to immunolabeling for fluorescence and CLSM (Confocal Laser Scanning Microscope) examination of the SCS8 aerogel and control sample. Both fluorescence and confocal examination combined, when possible, fluorescence and Nomarski modes in order to acquire both material and growing cells.

#### 2.7.2. Actin Cytoskeletal Organization and Focal Adhesion Development

At the end of each experiment, cells were washed twice with prewarmed phosphate-buffered saline (PBS), pH of 7.4, and fixed with 3.7% paraformaldehyde at room temperature, washed, and then permeabilized with 0.1% Triton x-100. After washing, cells were preincubated with 1% bovine serum albumin (Sigma) in PBS for 20 min prior to cell immunolabeling for actin cytoskeleton with rhodamine-phalloidin (Sigma) and monoclonal anti-vinculin FITC conjugate (Sigma). After 20 min, SCS8 aerogels and control samples were rinsed with prewarmed PBS prior to mounting with Hard Set Vectashield with DAPI^®^ (Vector, Burlingame, CA, USA). The measurement of FA (Focal Adhesion) size and location was conducted for at least 10 cells on each substrate.

#### 2.7.3. Confocal Examination

Samples were visualized using an Olympus confocal microscope. At least five samples were analyzed for each group to assess the surface influence on the cytoskeletal organization, focal adhesion number, development, and cell morphology. Images were collected and processed using imaging software. At least 50 cells per sample were analyzed. Samples were exposed to the lowest laser power that was able to produce a fluorescent signal for a time interval not higher than 5 min to avoid photobleaching. A pinhole of 1 Airy unit was used. Images were acquired at a resolution of 1024 × 1024.

#### 2.7.4. Image Analysis

To analyze the differences in focal adhesion number between different sample groups, images were collected and processed. Area, perimeter, roundness, circularity, solidity, and axial ratio were analyzed as shape variables. Sample images were collected as frames obtained at 40× magnification and processed using Image J software (NIH, http://rsb.info.nih.gov/ij).

All experiments were repeated in triplicates unless otherwise stated. All data were SPSS analyzed and expressed as the mean ± standard deviation. Once normality and homoscedasticity were confirmed, the difference between the mean values was analyzed using a two-tailed Student’s t-test and one-way analysis of variance. Statistical significance was defined as *p* < 0.05.

## 3. Results and Discussion

### 3.1. Textural Characterization

The textural parameters are measured by N_2_ physisorption. In all cases, type IV isotherms are obtained, which indicates the existence of a mesoporous entanglement that is CS content-dependent and generated by the high molar ratio of H_2_O:TEOS used, 30:1. 

**Figure 2:**
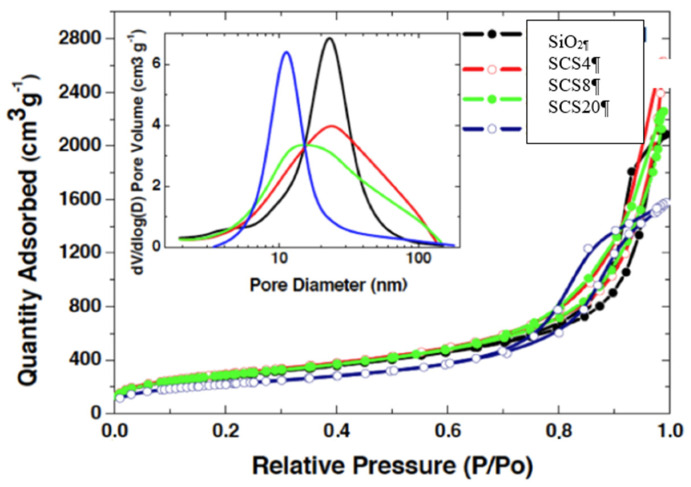
N_2_ physisorption isotherms of the outlined aerogels. Inset, pore size distribution (PSD) from the BJH as pore size distribution model [10].

As shown in Figure 2, there are two types of curves: one where the proportion of CS is low (SCS4 and SCS8), in which the curve does not level off at relative pressure close to 1, presenting hysteresis loop of type H3, characteristic of poor connectivity with pores in slit-like form. The second hysteresis loop (for pure silica (TEOS) and higher CS content (SCS20)) is type H1, which is characteristic of cylindrical pore geometry and narrower PSD (Pore Size Distribution), but good pore connectivity (inset Figure 2). Specific surface area and density decrease with the CS content (Table 1), while the porous volume and pore size do not present a defined behavior and seem to depend on the synthesis steps. Therefore, the results reveal the distorting influence of the CS on the aerogel network.

### 3.2. Thermal Analysis

**Figure 3:**
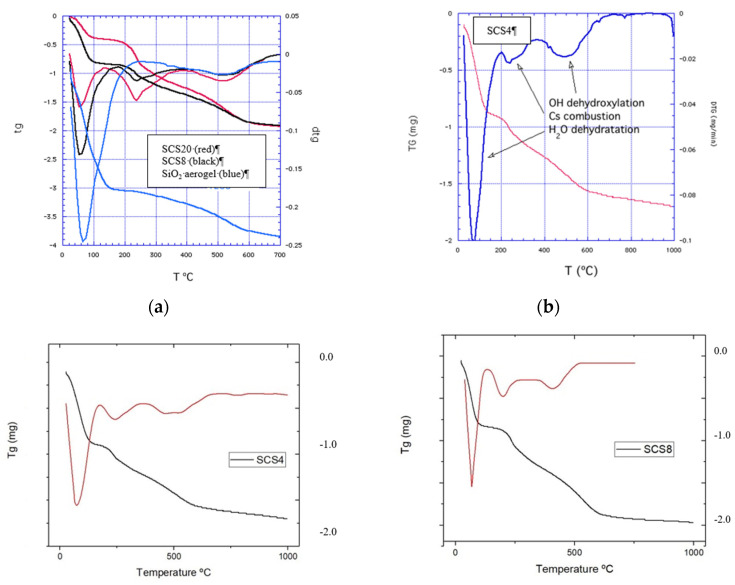

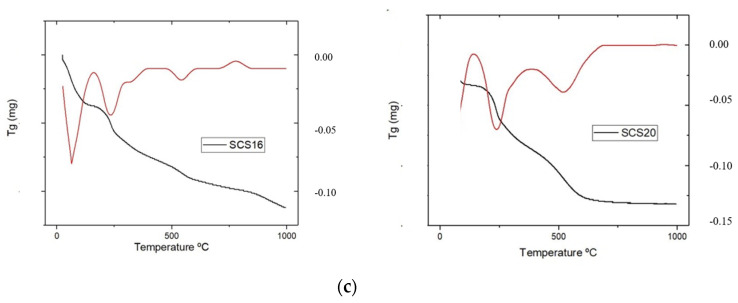
(**a**) TG and DTG of SCS20, SCS8 aerogels, and pure silica from TEOS. (**b**) TG and DTG of SCS4 aerogel with the three different weight drops. (**c**) TG (black) and DTG (red) curves of the SCS4, SCS8, SCS16, and SCS20 aerogels.

The properties of silica are determined by the concentration and geometrical distributions of silanol groups on the surface as well as by the presence of siloxane bridges. The pure silica aerogel (SiO_2_) shows two weight drops during the thermal scanning (Figure 3a). The first from 25 to 190 °C, accounting for the dehydratation process and accounting for a hydrophilic surface. A second drop is apparent from 400 to 700 °C due to the dehydroxylation of the silanols on the surface; later, weight loss corresponds to internal silanol groups.

**Figure 4:**
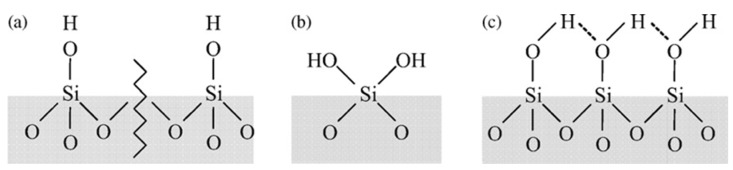
Schema of the silanol groups on the surface, (**a**) isolated, (**b**) geminal, and (**c**) vicinal.

As can be seen in Figure 3a,b, for composite aerogels, three processes occur during the thermal action on the silica surface—first, the dehydration (50–160 °C) that removes the physically adsorbed water (reversible by immersion in water); as a second step, the dehydroxylation by the removal of the vicinal silanol groups (Figure 4) and combustion of the CS (170–350 °C), absent in the pure silica aerogel (blue line). The dehydroxylation of the isolated –OH groups (Figure 4) takes place in a third step (400–700 °C) [24,25].

The thermogravimetric signals and its derivatives in relation to the time are shown in Figure 3c. In the case of the CS samples, three regions can be observed—dehydratation up to 200 °C, CS combustion with a peak at 240 °C, and dehydroxylation up to 700 °C coincident with the same process in pure silica aerogel. In the case of lower CS contents, the same signal can be discerned, as shown in Figure 3b for SCS4 aerogel.

The corresponding weight loss steps in the thermograms are used to estimate the hydroxyl content on the aerogel surface [25,26,27] using the relation:

(3) $$OH\left(nm^{-2}\right)=\frac{2\Delta mN_{A}}{100\cdot M_{w}\;S_{BET}}\cdot 10^{-18}$$

where Δ*m* is the weight loss between the corresponding temperatures on the thermogram, *N_A_* is the Avogadro number, *M_w_* is the molecular weight of water, and *S_BET_* the specific surface area of the aerogel. Factor 2 in the previous Equation (3) indicates that each water molecule consumes two hydroxyl groups to create a siloxane bridge, increasing the aerogel network. Factor 1 in Equation (3), used for the dehydration process, will be considered when only one water molecule is adsorbed by one –OH group. These silanol groups are distributed into three types—isolated free silanols (a) consist of one hydroxyl group per silicon atom, geminal silanols (b) consist of two hydroxyl groups attached to one silicon atom, and vicinal silanols (c), where the hydroxyl groups of adjacent Si atoms are linked through hydrogen bridges. In the case of curved surfaces, either concave or convex will affect the hydroxyl group’s content.

Table 2: From 400 to 700 °C (column 3), above CS decomposition temperature (200–400 °C), the weight loss is only due to the dehydroxylation of the silanol groups from the silica surface; smaller values indicate more adsorbed water on the structural CS hydroxyl groups as a solvation layer, that is, one water molecule for isolated silanol OH and one water molecule for two geminal or vicinal silanols. So, the difference between the two values (column 4-column 3) must correspond to the hydroxyl groups in CS biopolymer; the results seem to indicate a significant contribution of the hydroxyl groups at low CS content.

As can be inferred from this result, CS has little influence on the hydroxyl group content; it even causes its decrease. The hydroxyl contents of these composites are below the value of Kiselev–Zhuravlev constant [28], ranging from 4.6–4.9 OH/nm^2^. The higher values of the –OH groups (column 4) point out a multilayer formation of the absorbed water on the surface (first peak in DTG Derivative ThermoGravimetrydiagrams).

### 3.3. FTIR Spectra

**Figure 5:**
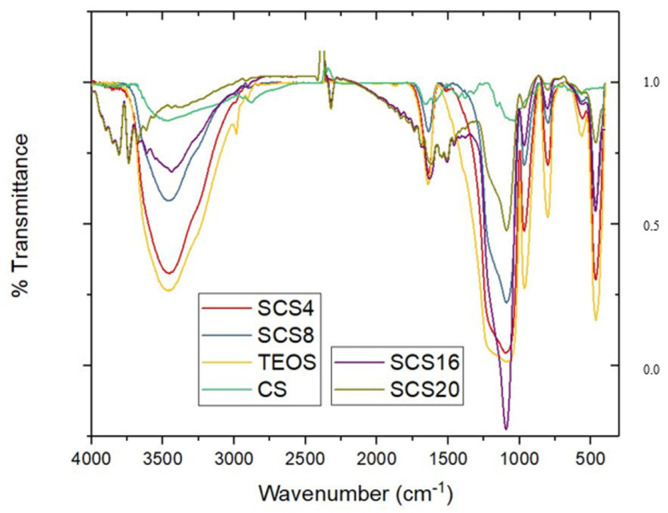
FTIR spectra of the outlined aerogels. CS (green) and TEOS (SiO_2_) (yellow) are included as a reference. Transmittance is in arbitrary units.

In all spectra as shows in Figure 5, the peaks corresponding to the structural bonding of silica at 467 cm^−1^ of the bending –O–Si–O–, at 800 and 965 cm^−1^ of the stretching Si–O–Si and Si-OH, respectively, are apparent. At higher frequency, the peak of the tetrahedra bond at ~1070 cm^−1^ (TO, transverse optical) and its high-frequency shoulder (LO, longitudinal optical at 1190 cm^−1^), which corresponds to the asymmetrical vibration of the longitudinal stretching of the Si–O–Si bonds, appears, and its decrease is apparent with the CS content. These peaks are concerned with strains on the Si–O–Si bonds and influence the angle and bond length. Hydroxyl groups CS content dependence is resolved in the broad-band ranging from 3000 to 3700 cm^−1^, related to the hydrogen bonds involving –NH_2_ and –OH groups and the vibrations corresponding to the isolated, geminal, and vicinal silanol groups.

### 3.4. Mechanical Properties

**Figure 6:**
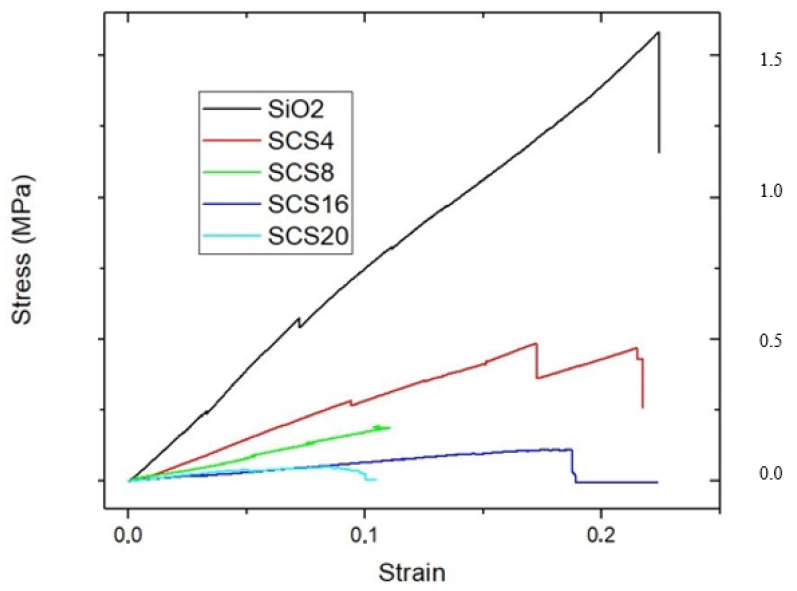
Stress/strain curves to failure of the outlined aerogels.

Figure 6 shows the stress/strain curves from uniaxial compression of aerogels. All the curves feature a very similar elastic response up to the fracture point without showing any plastic behavior, giving a compressive strength when the materials fail completely. This description corresponds to a viscoelastic behavior, which is known to be a time-dependent process and to exhibit hysteresis upon cyclic loading before rupture. The Young’s module decreases with the CS content as a consequence of the silica tetrahedra distortion produced by the interconnection of biopolymer units in agreement with the decrease of the LO vibrational IR shoulder (Table 3, Figure 4).

The compressive strength shows some increase with the influence of the biopolymer, and the deformation to fracture increases from TEOS aerogel to SCS4. Besides, a severe drop in the area under the curve indicates a decrease in the toughness, the ability to resist the fracture. This last effect confirms the loss of the pore interconnection, in agreement with the isotherms hysteresis loop (Figure 2). A decrease in Young’s modulus runs parallel with the CS content, which indicates a stiffness loss.

### 3.5. Water Uptake

**Figure 7:**
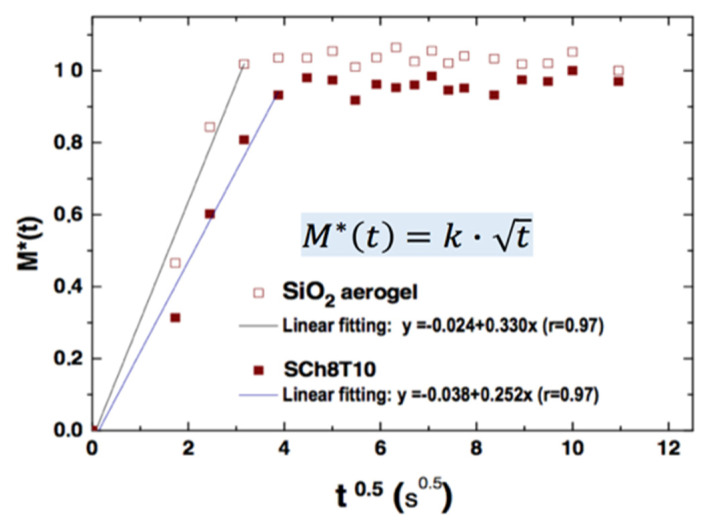
Water diffusion process inside the aerogel with (closed red) and without (open red) CS.

The water absorption behavior of the hybrid aerogels is evaluated by the representation of the normalized absorption mass ratio *M*(t)/*M*(∞) vs. *t*^0.5^ (shown in Figure 7), *M*(∞) being the normalized weight of samples after liquid saturation.

The good linear fit confirms that the process features a fickian diffusion behavior, and then a swelling of the polymer network can be expected as a key process in the bioactivity behavior.

### 3.6. Bioactivity

The aerogels are immersed in SBF to test their bioactivity. After two weeks, a layer of HAp microcrystals develops on the surface. The Ca/P molar ratio at different times is shown in Table 4. In relation to HAp crystals with a stoichiometric Ca/P molar ratio of 1.67, there is an apparent trend to grow more carbonated HAp crystals. In two weeks, crystals are calcium-deficient HAp and become carbonated apatite (CAp). The biocompatibility of CAp can be attributed to the tendency of the carbonate to reduce crystallinity within the apatite structure, being more biodegradable, thereby increasing its solubility, which enhances bone repair [28]. After four weeks, the structure attains the Ca/P ratio of tetracalcium phosphate (TCP).

This result asserts the bioactive behavior of the aerogel. On the other hand, the aerogels are ready to be implemented with osteoblasts cells in order to evaluate cell response at the initial steps of cell /material contact, cell adhesion, and focal adhesion development prior to evaluating a possible osteoinductive role of the materials. Osteoinduction refers to the process by which a tissue, or product derived from it, causes a second undifferentiated tissue to differentiate into bone [29,30].

**Figure 8:**
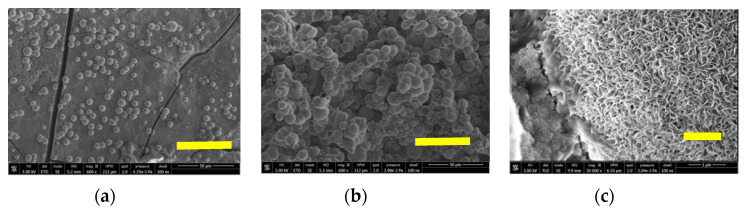
SEM micrographs of SCS8 aerogel after two weeks (**a**) and four weeks (**b**) immersion in SBF, right bottom bar 50 μm. Micrograph (**c**) shows details of the needle-like surface crystallites that form the spherulite, right bottom bar 1 μm.

The hydroxyl content of these aerogels, shown in Table 2, induces apatite nucleation, and the released Ca^++^, Na^+^, K^+^ cations from the SBF accelerate this apatite nucleation by increasing the ionic activity product, as the body fluid is highly supersaturated with respect to the HAp [31]. The formation of an amorphous calcium phosphate layer permits further growth by its crystallization as spherulites composed of needle-like apatite nanocrystals with preferential orientation along the c-axis. The SEM micrographs in Figure 8a–c show the HAp crystals as spherulites, whose particle size has grown from 6 to 12 μm covering all surfaces.

**Figure 9:**
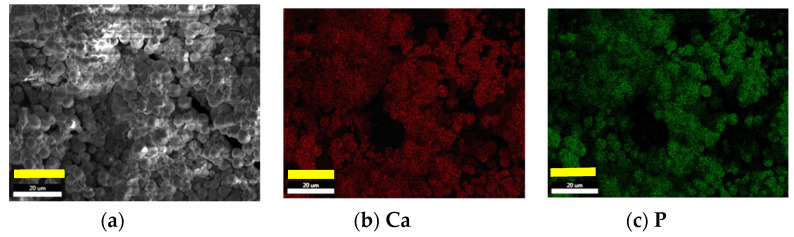
SCS8 aerogel after four weeks of immersion in SBF (**a**). Maps from EDX spectra of the ions distributions for the calcium (**b**) and phosphorus (**c**). (left bottom bar 20 μm).

Energy-dispersive X-ray spectroscopy is carried out on the aerogels with HAp crystal on the surface. The analysis permits knowing the molar ratio of the different ions forming the crystals. In this way, maps of the different ions can be registered, as shown in Figure 9. As observed, the maps of both ions are coincident, proving the apatite crystal nature and homogeneity.

### 3.7. Assessment of Osteoblast Behavior

**Figure 10:**
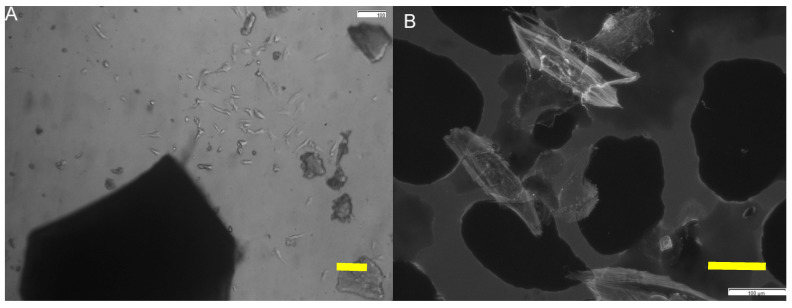
HOB^®^ osteoblasts grown on SCS8 sample after 24 h in culture (**A**) and examined with a phase-contrast microscope, showing cell elongation, organization, and polarization towards aerogel particles. In (**B**), cells are imaged in the fluorescence mode by the confocal microscope, showing osteoblasts polarization and approach to the material. Grayscale allows for the identification of actin immunolabeling (white) and shows how cells start to colonize the material surface (black). (right bottom bar 100 μm).

As shown in Figure 10, normal human osteoblasts start to polarize after 24 h in culture, and after 48 h, they approach the material tethering with filopodial and lamellipodial emissions (Figure 10a). Once reached, the material is covered by migrating osteoblasts from 48 h onwards (Figure 10b). Cell viability at seeding is up to 98%. No significative apoptotic phenomena are detected either in the control or experimental groups.

**Figure 11:**
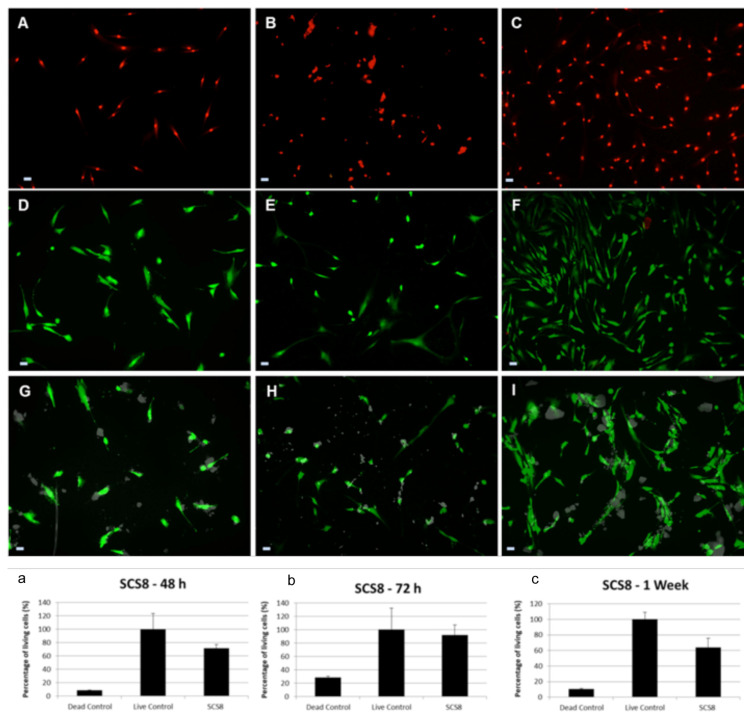
Live/dead staining of HOB^®^ osteoblasts growing on: (**A**) Glass, 48 h in culture. Negative control obtained after 70% ethanol exposure for 30 min before staining; (**B**) Glass, 72 h in culture, negative control; (**C**) Glass, one week in culture, negative control; (**D**) Glass, 48 h in culture. Positive control; (**E**) Glass, 48 h in culture. Positive control and (**F**) Glass, one week in culture. Positive control; HOB cultures with SCS8: (**G**) SCS8 after 48 h in culture, (**H**) SCS8 after 72 h in culture, and (**I**) SCS8 after 1 week in culture. Live cells appear green; nuclei of dead cells fluoresce red and SCS8 in gray. Scale bar represents 50 μm. Shown in the graph, the percentage of live cells. Live/dead staining of HOB^®^ osteoblasts growing on glass (controls) or SCS8 after: (**a**) 48 h in culture; (**b**): 72 h in culture, and (**c**) 1 week in culture. Negative control (dead control) is obtained after 70% ethanol exposure for 30 min before staining. Reference value (100%) for live cells (live control) obtained from HOB^®^ cells grown on glass without biomaterial, under optimal conditions and at the indicated times. Quantification analysis in 10 images per experiment (n = 3) and expressed as mean ± SEM.

Live/dead staining has revealed that the majority of cells are in a viable state (green) at all time points, with only a few dead cells (red), as shown in Figure 11. Live dead assay is performed to assess cell viability and growth. HOB cells, grown on SCS8 for 48 h, show 71.458% living cells, while the dead control group show 8.53%. After 72 h, living cells reach 92.174%, while in the negative control, it is up to 28.476%. After 1 week in culture, 63.658% living cells are found in SCS8, and 10.547% in the negative control. Due to the presence of biomaterials, cells migrate to reach the aerogel and then adhere to the surface, while a lower percentage of cells adhere to the glass bottom of the well. Cells growing on the material develop more efficient cell adhesion complexes than those in the positive control groups as initial steps for differentiation. Ongoing studies in our lab are now on the way to demonstrate this aspect. In control groups, cells expand and proliferate without significant migration anywhere.

The seeding has resulted in a regular dispersion of cells, which initially approach the material pieces, and after 72 h in culture, start to differentiate into branched cells, with osteocyte-like prolongations approaching the material present in the well, displaying long, thin cell processes extending from a small, rounded cell body throughout the experiment duration, as shown for 1-week cells, which proliferate and migrate to cover the scaffold surface.

**Figure 12:**
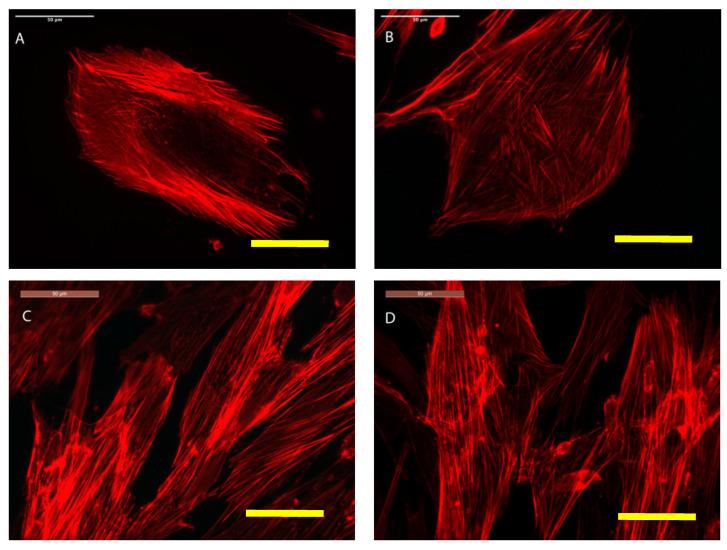

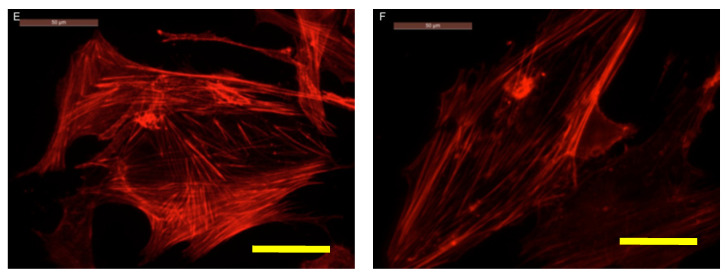
HOB^®^ osteoblasts grown on the glass are used as a reference control, shown in (**A**,**B**) after 48 h in culture, and examined with a confocal microscope. HOB^®^ osteoblasts grown on SCS8 sample after 48 h (**C**,**D**) and 72 h (**E**,**F**) and examined with a confocal microscope. In red, rhodamine-phalloidine immunolabeled actin cytoskeletal fibers showing polarization to material and actin stress fibers development. In all cases yellow bar 50 μm.

As shown in detail (Figure 12), actin-immunolabeled osteoblasts are efficiently grown on the SCS8 aerogel with cell polarization, elongation, and significant expression of stress fibers, which has been described previously by us and others as a marker of cell adhesion and differentiation due to their role in cytoskeletal rearrangement [32,33,34].

**Figure 13:**
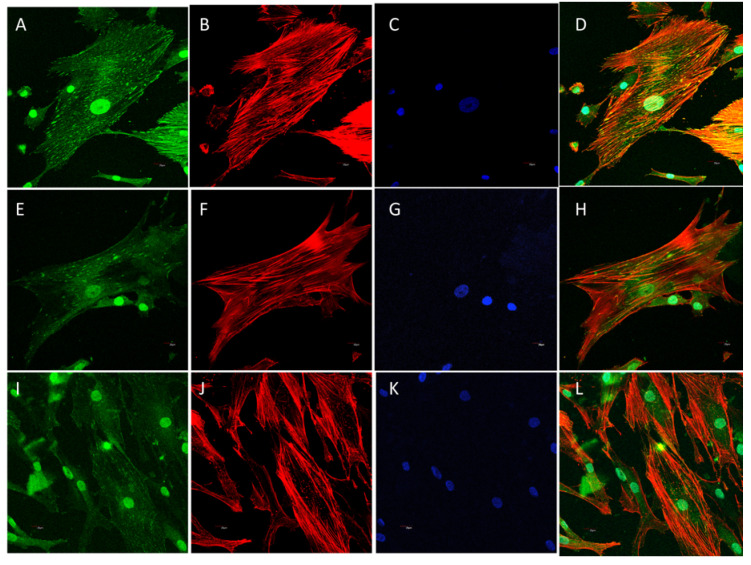
Representative images of HOB^®^ cells grown on SCS8 aerogel and immunolabeled for actin cytoskeleton (rhodamine-phalloidine, red) and focal adhesion development (vinculin, green). Nuclei are identified with DAPI. Cells immunolabeled after 48 h in culture (**A**–**D**); 72 h in culture (**E**–**H**); 1 week (**I**–**L**).

**Figure 14:**
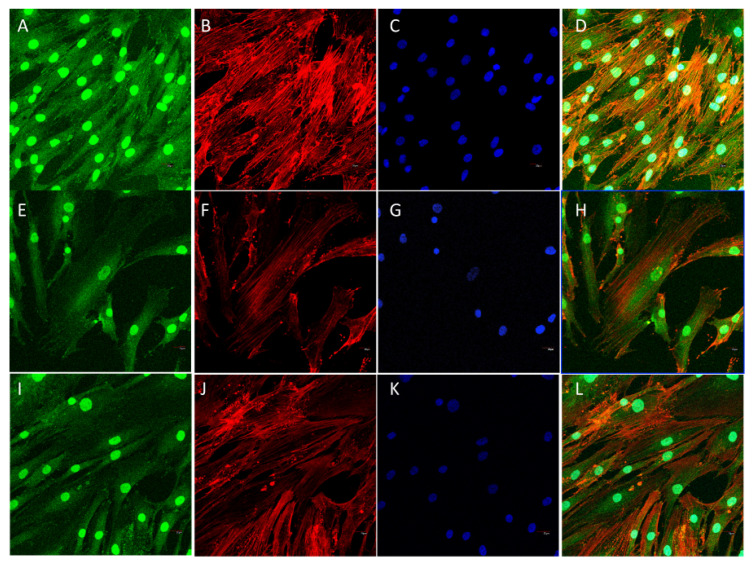
Representative images of HOB^®^ cells grown on glass bottom wells and employed as controls immunolabeled for actin cytoskeleton (rhodamine phalloidine, red) and focal adhesion development (vinculin, green). Nuclei are identified with DAPI. Cells immunolabelled after 48 h in culture (**A**–**D**); 72 h in culture (**E**–**H**); 1 week (**I**–**L**).

**Figure 15:**
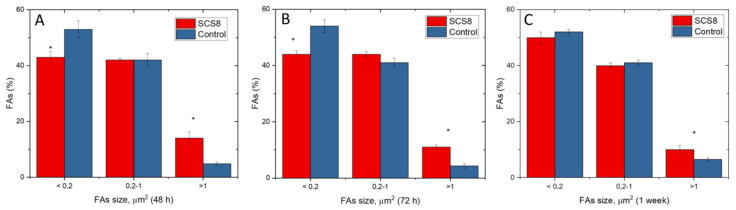
Percentage of FAs according to size. Comparative analysis between cells grown on SCS8 and control groups at (**A**): 48 h in culture; (**B**): 72 h hours in culture; (**C**): 1 week in culture. Data are quantified in Image J (*); *p* < 0.05.

**Figure 16:**
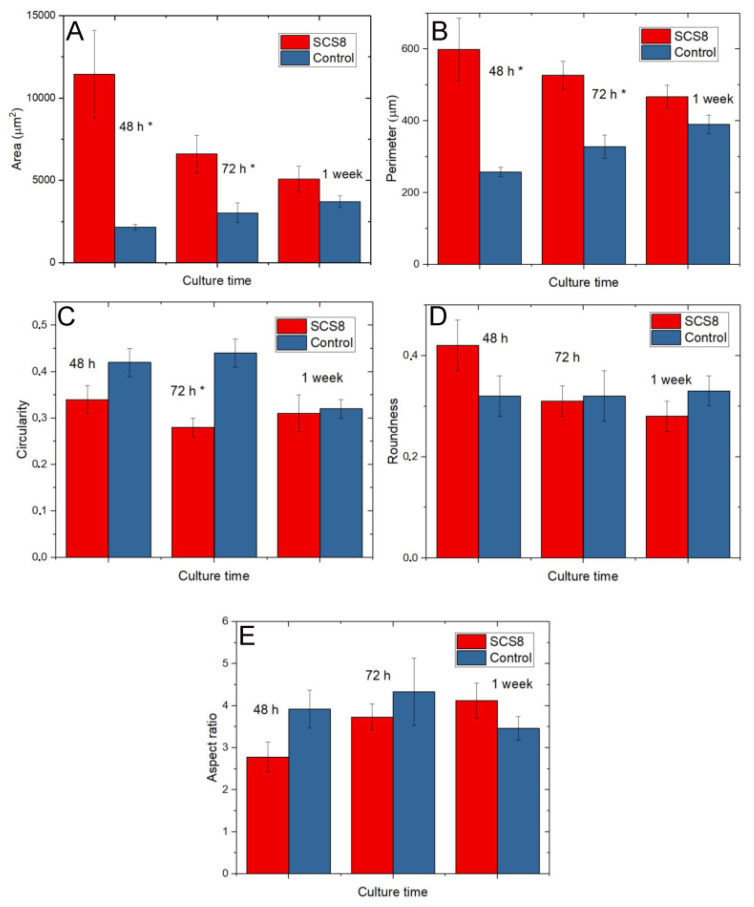
Shape variables. (**A**) Quantification of cellular spread area, (**B**) Quantification of cellular perimeter, (**C**) Quantification of cellular circularity, (**D**) Quantification of cellular roundness, (**E**) Quantification of cellular aspect. Data quantified in Image J (*); *p* < 0.05.

On the spread of cells, quite a number of FAs are located in the center of the cell, as shown in the controls, with circular morphology and small size. There is FA distribution in cells grown on the biomaterial changes, with a higher assembly near the periphery of elongated FAs in which size increases in the lamellipodia and filopodial tips. The differences are observed initially after 48 h in culture, and FAs reorganization increases with time, as well as the differences between experimental and control cells. Together with FA reassembly, the actin cytoskeleton is also reorganized, and stress fibers start to appear as long and aligned actin bundles between big, elongated FAs, clearly after 72 h, together with an increase in the number and size of mature FA compared to the control in all experimental times, as shown in Figure 13, Figure 14 and Figure 15 and Table 5. Filopodial and lamellipodial prolongations increase in number and size. Changes in the cell morphology driven by actin cytoskeletal fibers appear to be evident after 1 week (Figure 16), where stress fibers organize in the periphery of well orientated and non-migrating cells.

The comparative analysis of FA maturation based on FA size has revealed significant differences between experimental groups and control groups. Control cells present a higher percentage of punctate, non-mature, i.e., <0.2 μm^2^ FAs than experimental groups, both at 48 and 72 h. In contrast, the percentage of FAs sized >1 μm^2^ is significantly higher in the SCS8 group than in control cells at these experimental times. After 1 week in culture, the percentage of FAs > 1 μm^2^ remains significantly higher than in controls.

Significant differences in spread area are found between cells grown on SCS8 both after 48 h and 72 h in culture. Furthermore, the variable cell perimeter also presents significant differences after 48 and 72 h in culture in the presence of aerogel. Additionally, after 72 h in culture, significant differences in circularity are found in control cells and cells grown in the presence of SCS8 (Table 6).

Bone has the capacity throughout life to regenerate in response to several injuries in a process largely mediated by osteoblasts, which appear at injuries, divide, and secrete new bone matrix components. During osteogenesis and regeneration processes, osteoblasts differentiate into osteocytes, which are capable of synthesizing both the osteoid and fibers that make up the extracellular matrix. Osteocytes represent the final differentiation step in the osteoblastic lineage. Osteoblasts lose a large part of their cell organelles but gain long, thin, and branched cell processes by which the cells remain in contact with neighboring osteocytes, capillaries, and osteoblasts lining the bone surface. Osteogenic cells have a highly developed cytoskeleton, and it is known that their differentiation is regulated in part through mechanical forces imposed by their surrounding environment [35,36,37].

When a biomaterial is exposed in vitro to a supplemented cell culture medium, proteins, mainly fibronectin and vitronectin in serum, start to adhere to the surface by means of non-covalent reactions. Specific binding sites on these proteins are recognized by cell membrane integrins. Then, the integrins assemble in the so-called focal adhesion (FA) complexes and couple to filamentous actin (F actin), using linker proteins, such as vinculin [38,39].

Focal adhesions are formed during initial cell adhesion and then are constantly assembled and disassembled as the cell moves. During this period, they also act as mechanosensors for both biochemical and biophysical properties of the material, and concordant cytoskeletal modulation takes place together with changes in FA size and number both in stationary and migrating cells [38]. FA transmits the force generated by different actin networks and interacts with the retrograde flow of the lamellar actin network in many cell types with actomyosin bundles called stress fibers [40]. Various studies have demonstrated both the effect of extracellular mechanics on osteogenic differentiation and how the internal machinery of the cell, consisting of tensile (actin) and compressive (microtubule) elements, and FA attachment complexes play an important role in the translation of extracellular forces and alter fundamental cell behaviors, such as viability and migration [37,38,41,42].

Depending on the cellular motility, two different types of FA have been described: In the case of migrating cells, small focal adhesions (also named adhesion complexes) appear on the filopodia or lamellipodia leading edges, mostly composed of integrins interacting with the actin cytoskeleton due to the presence of talin and vinculin. They are also called Nascent punctate nanoscale (0.2 μm^2^). These nascent adhesions either undergo rapid turnover within the lamellipodium or mature into larger, elongated FAs under the influence of myosin-dependent cytoskeletal tension. Mature, large (1.0–10 mm^2^) FAs anchor cells to the substrate to maintain cell morphology and tensional homeostasis. In non-migrating cells, mature large FAs can be formed or even localized in the cell body during migration. They are predominantly composed of vinculin, paxilin, α actinin, and FAKs (Focal Adhesion Kinases) [35,36,43,44].

To improve new bone growth, careful tailoring of the scaffold is an important step for successful bone tissue engineering applications [45]. As a natural polymer, chitosan offers an adequate set of characteristics for developing advanced functions, such as biocompatibility, biodegradability, hydrophilicity, and non-toxicity. FAs play a role in the process of surface recognition, with subsequent cell adhesion and mechanotransduction-induced changes in osteoblastic cells. When the nanoscaled biomaterial surface is chemically attractive, additional cues for cell positioning, spreading, and differentiation can be achieved [32,35,46,47,48]. The improved characteristic in our scaffold properties, which are appropriate porosity, pore structure, and pore size of the scaffold, have demonstrated a positive effect on cell adhesion, with focal adhesion recruitment and development and initial osteoblasts differentiation.

Thus, both aerogel physicochemical properties and aerogel surface configuration are found to be of importance not only for osteoblasts guidance, with cell elongation and filopodial and lamellipodial emissions looking for focal adhesion points [35,38,41], but also for inducing stress fibers formation, which is indicative of the presence of active force-generating actin for satisfactory cell anchorage with a decrease of cell migration and described as the initial point for osteoblast differentiation for bone formation [49,50,51].

## 4. Conclusions

Aerogels of the SiO_2_/CS system are obtained by the sol-gel method, promoting the hydrolysis reaction with high power ultrasounds. These materials, classified as hybrids organic/inorganic, have been used to mimic human bone (HAp/collagen) as well as in soft tissues.

Monolithic hybrids aerogels are obtained with a high specific surface area (800–1100 m^2^ g^−1^) and large porous volume (2.5–4.0 cm^3^ g^−1^). The IR spectra confirm the silica matrix structure crosslinking by the CS biopolymer chains (1100–1200 cm^−1^) and reveal the surface hydroxyl groups (3000–3700 cm^−1^). The aerogels are elastic with a low Young modulus, which is CS content-dependent.

CS controls the hydroxyl density on the surface (–OH nm^−2^), covering the surface acting as nucleation sites for the HAp crystal growth. The supersaturated SBF, with respect to apatite, accelerates its growth. In two weeks, 6 μm crystal size and Ca/P molar ratio of 1.67 are observed.

Bioactivity and degradation rates are tested prior to cellular assays. Normal human HOB^®^ osteoblasts are grown on the silica/CS aerogel. In order to identify the osteoconductive properties of the aerogels, morphological alterations, cell viability and proliferation, and initial cytoskeletal changes as markers of cell adhesion are assessed after 48 and 72 h in culture.

The results presented herein point to the positive effect of the designed aerogel for bone tissue engineering both in the osteoconductive and osteoinductive roles.

## Figures and Tables

**Table 1:** Textural parameters of the silica/CS aerogels; the number in the sample code refers to the CS wt% content. Column 2 is the result of the chemical analysis.

Aerogels	Real CS%	S (BET) m^2^/g	ρ (g/cm^3^)	Vp (cm^3^/g)	Pore Size (nm)
SiO_2_ aerogel	0	994	0.176 ± 0.011	3.195	12.854
SCS4	1.67 ± 0.03	1072	0.199 ± 0.009	3.742	13.950
SCS8	6.53 ± 0.04	1047	0.161 ± 0.033	2.936	11.211
SCS16	no data	840	0.136 ± 0.003	2.964	14.108
SCS20	10.29 ± 0.02	786	0.144 ± 0.007	2.401	12.213

**Table 2:** Hydroxyl groups content on the aerogel surface from TGA weight drops. SBET (specific surface area from BET model).

Aerogel (CS)	SBET (m^2^g^−1^)	OH nm^−2^ (400–700 °C)	OH nm^−2^ Geminal and Vicinal (25–190 °C)
SiO_2_ aerogel	994	5.12	13.86
SCS4	1072	1.81	4.74
SCS8	1048	3.26	4.78
SCS16	840	2.39	3.98
SCS20	786	5.45	3.48

**Table 3:** Young’s elastic modulus values of the outlined aerogels.

Aerogel	Young’s Modulus (MPa)
SiO_2_ aerogel	11.57 ± 3.84
SCS4	2.61 ± 0.56
SCS8	0.95 ± 0.13
SCS16	0.66 ± 0.01
SCS20	0.72 ± 0.16

**Table 4:** Ca/P molar ratio at different immersion times soaked in SBF.

Time in SBF	SCS8
1 week	0.48
2 weeks	1.57
4 weeks	1.99

**Table 5:** Percentage of focal adhesions compared by size and time. Statistically significant differences are labeled with an asterisk (*), *p* < 0.05. (M = mean and SE = standard error).

	SCS8 FAs 48 h	Controls FAs 48 h	SCS8 FAs 72 h	Controls FAs 72 h	SCS8 FAs 1 Week	Controls FAs 1 Week
%FA < 0.2 μm^2^	M = 43.777% SE = 2.075 (*)	M = 53.073% SE = 2.918 (*)	M = 44.464% SE = 1.319 (*)	M = 53.931% SE = 2.263 (*)	M = 49.52% SE = 5.26	M = 52.42% SE = 3.62
%FA 0.2–1 μm^2^	M = 42.02% SE = 1.89	M = 42.18% SE = 5.73	M = 43.99% SE = 2.66	M = 41.72% SE =5.09	M = 40.52% SE = 2.56	M = 41.14% SE = 2.92
%FA > 1 μm^2^	M = 14.203% SE = 2.189 (*)	M = 4.741% SE = 0.617 (*)	M = 11.546% SE = 0.750	M = 4.343% SE = 0.859 (*)	M = 9.963% SE = 1.519 (*)	(M = 6.438% SE = 0.604 (*)

**Table 6:** Shape parameters by time and group. Statistically significant differences are labeled with an asterisk (*), *p* < 0.05. (M = mean and SE = standard error).

	SCS8 48 h	Controls 48 h	SCS8 72 h	Controls 72 h	SCS8 1 Week	Controls 1 Week
Spread area (μm^2^)	M = 11439.794 SE = 2672.354 (*)	M = 2163.724 SE = 157.954 (*)	M = 6604.080 SE = 1138.230 (*)	M = 3030.626 SE = 602.313 (*)	M = 5079.371 SE = 791.598	M = 3700.125 SE = 355.046
Cell perimeter(μm)	M = 599.210 SE = 1138.230 (*)	M = 258.910 SE = 13.300 (*)	M = 527.810 SE = 38.884 (*)	M = 328.400 SE = 32.1 (*)	M = 467.580 SE = 32.461	M = 390.394 SE = 26.502
Circularity	M = 0.339 SE = 0.032	M = 0.421 SE = 0.028	M = 0.276 SE = 0.032 (*)	M = 0.437 SE = 0.024 (*)	M = 0.313 SE = 0.040	M = 0.321 SE = 0.023
